# Rich-Club Analysis of Structural Brain Network Alterations in HIV Positive Patients With Fully Suppressed Plasma Viral Loads

**DOI:** 10.3389/fneur.2022.825177

**Published:** 2022-06-24

**Authors:** Xire Aili, Wei Wang, Aidong Zhang, Zengxin Jiao, Xing Li, Bo Rao, Ruili Li, Hongjun Li

**Affiliations:** ^1^Beijing Advanced Innovation Center for Biomedical Engineering, Beihang University, Beijing, China; ^2^Department of Radiology, Beijing Youan Hospital, Capital Medical University, Beijing, China; ^3^Department of Radiology, Zhongnan Hospital of Wuhan University, Wuhan University, Wuhan, China; ^4^Department of Radiology and Nuclear Medicine, Xuanwu Hospital, Capital Medical University, Beijing, China

**Keywords:** HIV, structural connectivity, DTI, graph theory, rich club

## Abstract

**Objective:**

Even with successful combination antiretroviral therapy (cART), patients with human immunodeficiency virus positive (HIV+) continue to present structural alterations and neuropsychological impairments. The purpose of this study is to investigate structural brain connectivity alterations and identify the hub regions in HIV+ patients with fully suppressed plasma viral loads.

**Methods:**

In this study, we compared the brain structural connectivity in 48 patients with HIV+ treated with a combination of antiretroviral therapy and 48 healthy controls, using diffusion tensor imaging. Further comparisons were made in 24 patients with asymptomatic neurocognitive impairment (ANI) and 24 individuals with non-HIV-associated neurocognitive disorders forming a subset of HIV+ patients. The graph theory model was used to establish the topological metrics. Rich-club analysis was used to identify hub nodes across groups and abnormal rich-club connections. Correlations of connectivity metrics with cognitive performance and clinical variables were investigated as well.

**Results:**

At the regional level, HIV+ patients demonstrated lower degree centrality (DC), betweenness centrality (BC), and nodal efficiency (NE) at the occipital lobe and the limbic cortex; and increased BC and nodal cluster coefficient (NCC) in the occipital lobe, the frontal lobe, the insula, and the thalamus. The ANI group demonstrated a significant reduction in the DC, NCC, and NE in widespread brain regions encompassing the occipital lobe, the frontal lobe, the temporal pole, and the limbic system. These results did not survive the Bonferroni correction. HIV+ patients and the ANI group had similar hub nodes that were mainly located in the occipital lobe and subcortical regions. The abnormal connections were mainly located in the occipital lobe in the HIV+ group and in the parietal lobe in the ANI group. The BC in the calcarine fissure was positively correlated with complex motor skills. The disease course was negatively correlated with NE in the middle occipital gyrus.

**Conclusion:**

The results suggest that the occipital lobe and the subcortical regions may be important in structural connectivity alterations and cognitive impairment. Rich-club analysis may contribute to our understanding of the neuropathology of HIV-associated neurocognitive disorders.

## Introduction

With the success of combination antiretroviral therapy (cART), human immunodeficiency virus positive (HIV+) patients no longer suffer from severe complications of the disease and have nearly the same life expectancy as healthy individuals without it. Before successful cART, HIV+ patients were at high risk for dementia. In the post-cART era, though the incidence of serious dementia has dramatically decreased, a milde form of cognitive impairment is known to persist in these individuals (1). The HIV Neurobehavioral Research Center- Revised American Academy of Neurology criteria provide a more precise subdivision of HIV-associated neurocognitive disorders (HAND) into asymptomatic neurocognitive impairment (ANI), HIV-associated mild neurocognitive disorder, and HIV-associated dementia (2). In the most recent meta-analysis on HAND, ANI was reported to be the most common form of cognitive impairment, accounting for 23.5% of cases worldwide (3). Although theoretically, as per its definition, ANI does not affect daily functioning, patients with ANI are still at risk of deterioration of neurological condition into further cognitive decline (4). In the pre-cART era, HAND was found to be highly related to plasma viral load and cluster of differentiation 4 (CD4) counts (5). With improved precision of the infectious diseases reporting system and positive treatment adherence of HIV+ patients, an increasing number of patients with HIV are generally on successful cART and have plasma viral loads under the detectable threshold (6). Therefore, HIV+ patients with fully suppressed viral loads are now frequent participants in clinical trials. Subtle alterations, including whole-brain white matter damage and cognitive impairments in psychomotor speed and executive functioning, have been detected in these patients (7, 8).

Diffusion tensor imaging (DTI) is a non-invasive imaging approach that can assess the white matter integrity and reveal the tractography (9). Along with disease severity and neurological impairment, HIV+ patients tend to have increased mean diffusivity (MD) and decreased fractional anisotropy (FA) (10), which may indicate the damage to white matter integrity (11). Previous studies on virally suppressed patients have demonstrated diffused white matter alterations (7). White matter alterations were also considered to be associated with cognitive deficits (12, 13). Basic DTI analysis, on the other hand, was unable to reveal structural connections between cortical brain regions (14). Therefore, using structural network analysis with the graph theory methodology could help us understand the connectivity alterations of brain regions. The whole brain can be considered as a single graph consisting of nodes linked by edges, with different brain regions being parceled as nodes and white matter tracts between them representing the edges (15–17). Previous structural network research showed that frontal and motor connections were compromised in older HIV+ patients (18), and young untreated HIV+ patients showed decreased efficiency, characteristic path length, clustering coefficient, and connection strength (19). Bell et al. (20) revealed a lower FA clustering coefficient and lesser MD nodal degree in the thalamus of HIV+ patients on cART. These studies demonstrated the structural connectivity impairments between brain regions, and they may serve as potential neuroimaging biomarkers for HAND.

The “hubs” refer to specific brain regions with a high node efficiency and degree centrality, a short path length, and low clustering. They are considered to play a central role in the network (21). “Rich-club” refers to the connections between hubs, which are greatly connected to each other rather than with low degree nodes (22). A previous study suggested that brain networks had a hierarchical and assortative property (23), and hub nodes, being the central core, could integrate information throughout numerous segregated brain regions for better cognitive processing, thereby it is important to investigate hub nodes (24, 25). Yadav et al. (26) reported that the structural network hubs in pediatric HIV+ patients are located in the left fusiform gyrus and the left precuneus. Previous research on Alzheimer's disease (AD) demonstrated that abnormal connections between rich-club nodes could reflect the progression of AD (27). To the best of our knowledge, the hub regions of HIV+ adults and the abnormal rich-club connections within the structural network of HIV+ patients have not been reported yet.

One limitation of these studies is that the viral loads in some patients were still detectable. We conducted the analysis on HIV+ patients with fully suppressed viral loads in the plasma. Furthermore, we conducted subgroup analyses on 24 patients with ANI and 24 non-HAND patients as a subset of HIV+ patients, with the hypothesis that there would be more alterations in patients with ANI. We aimed to reveal the structural network alterations in HIV+ patients and patients with pre-clinical cognitive impairments, thereby allowing us to understand the structural alterations occurring in patients with varying cognitive statuses. We explored the correlation between structural network alterations in cognitive performance and clinical variables. Understanding these changes can help us deepen our collective knowledge of the neurodegenerative mechanisms underlying HAND, enabling us to discover potential biomarkers for the early detection and prevention of this condition.

## Materials and Methods

### Participants

The HIV+ patients in this study were recruited from clinics in Beijing Youan Hospital, Capital Medical University, China. The study cohort consisted of 48 HIV+ patients (mean age 32.25 ± 5.67 years, 46 men, 2 women) and 48 healthy controls (mean age 34.52 ± 6.52 years, 44 men, 4 women); the two groups were matched for age and sex. Two subsets of the HIV+ patients' group were created, the ANI group (mean age 31.67 ± 5.47 years, 23 men, 1 woman) and the non-HAND group (individuals with a normal cognitive state; mean age 32.83 ± 5.92 years, 23 men, 1 woman). All participants signed written informed consent forms prior to participation, and the research protocol was approved by the Beijing Youan Hospital Ethics Committee. The inclusion criteria were as follows: age between 18 and 55 years; HIV+ patients on cART for over 3 months and complete suppression of HIV RNA in the plasma; availability of complete clinical records (e.g., CD4 counts, and CD4/CD8 ratio); a minimum education of at least ten years; and no contraindications to magnetic resonance imaging (MRI). The exclusion criteria were serious psychiatric conditions that could alter the cognitive performance (e.g., schizophrenia and bipolar disorder); other neurological disorders or the central nervous system (CNS) diseases; former traumatic brain injury plus loss of consciousness for over 30 min; opportunistic CNS infections or any tumors; and people with substance use disorder (e.g., alcohol, marijuana, and nicotine).

### Neuropsychological Testing

Eligible participants completed a multi-range standard battery of neuropsychological (NP) tests 2 h before the MRI that covered six cognitive territories: verbal fluency was tested by the Animal Verbal Fluency Test (AFT); attention/working memory was tested by the Wechsler Memory Scale (WMS-III), the Continuous Performance Test-Identical Pair (CPT-IP), and the Paced Auditory Serial Addition Test (PASAT); executive function was tested using Wisconsin Card Sorting Tests (WCST-64); memory and specifically learning and delayed recall was tested by the Hopkins Verbal Learning Test (HVLT-R) and the Brief Visuospatial Memory Test (BVMT-R); and information processing speed was tested by the Trail Marking Test A (TMT-A). Fine motor skills of both the dominant and non-dominant hands were tested using a grooved pegboard (28, 29). Additionally, participants were also assessed with short activities of daily living scale for evaluating cognitive difficulties in daily life and used self-assessed questionnaires for daily routine function (30). T-scores were calculated by adjusting for sex, age, and educational level, and domain-specific T-scores (average T-scores) were calculated afterward. Importantly, the diagnosis of ANI was made as per the Frascati criteria (2), including participants without cognitive difficulties in daily life but having scores of at least one standard deviation (SD) below the adjusted mean in not less than two cognitive territories.

### Image Acquisition

Brain images were acquired on a Siemens Trio 3 Tesla MR scanner (Siemens, Erlangen, Germany) using a 32-channel phased-array head coil. Participants were requested to close their eyes and remain still during the exam, and the head was immobilized with plastic pads. For DTI data, a single-shot echo-planar imaging sequence was used for acquisition. The DTI sequence parameters were as follows: time of repetition (TR) = 3,300 ms, echo time (TE) = 90 ms, matrix size = 128 × 128, slice thickness = 4 mm, field of view (FOV) = 230 × 230 mm, space resolution = 1.8 × 1.8 × 1.8 mm^3^, number of excitations = 3, and total acquisition time = 3.39 min. Further, 20 non-collinear diffusion sensitizing gradient directions (b = 1,000 s/mm^2^, and 1b = 0 s/mm^2^) were applied. Axial T1-weighted images (T1WI) in the 3D-Magnetization-Prepared Rapid Acquisition Gradient-Echo (MPRAGE) sequence with TR = 250 ms and TE = 2.46 ms were obtained: TI = 900 ms, filed of view = 250 × 250 mm, acquisition matrix = 256 × 246, number of slices = 176, slice thickness = 1 mm, flip angle = 9°, and voxel size = 1 × 0.977 × 0.977 mm^3^. The mean scan duration was 4 min 18 s.

### Image Preprocessing and Network Construction

All the data were preprocessed with Pipeline for Analyzing Brian Diffusion Images (PANDA) software (31). The exact preprocessing steps were as follows: conversion of all DICOM images into four-dimensional NIFTI format images, extraction of the whole brain tissues by removing all non-brain structures (e.g., the skull) to acquire the mask for the whole brain and to improve registration accuracy, eddy current distortion, and motion artifact correction (FSL; http://www.fmrib.ox.ac.uk/fsl). Each co-registered 3DT1-weighted image was further non-linearly normalized into the MNI-ICBM152 template afterward. Fractional anisotropy (FA) between the brain regions and spatial standardization were calculated. The Automated Anatomical Labeling (AAL) model was used to divide the brain into 90 brain regions; each brain region was regarded as a network node. Fiber assignment by continuous tracking (FACT) algorithm within Diffusion Toolkit (http://trackvis.org/dtk/) was used as a deterministic fiber tracking method to track the whole-brain fiber. A streamline was terminated based on the threshold value, which was set as FA <0.2 and tracking angle <45° (between two adjacent voxels). The FA-based interregional connection (FABIRC) was calculated as the average of the FA values of all contained streamlines, which formed the interregional connection. Inverse transformations were used to convert the AAL atlas from the MNI space to the DTI space. All brain regions were parceled into the following 10 different resting-state networks (RSNs): default mode network (DMN), salience network (SN), visual network (VN), sensory-motor network (SMN), primary auditory cortex (PAC), executive network (EXN), orbitofrontal cortex (ORB), olfactory cortex (OLF), thalamus (THA), and other (32, 33).

### Graph Theory

#### Global Properties of the Network

Graph theory analysis spotlights both global and regional aspects. We investigated parameters in all groups. The global clustering coefficient, characteristic path length, global efficiency, connection strength, and small-worldness (14) were computed. Details of these parameters can be found in the Supplementary Material.

#### Regional Network Characteristics

At the regional level, several regional parameters were computed: degree centrality, betweenness centrality, nodal shortest path length, nodal cluster coefficient, and nodal efficiency (34). Details of these parameters can be found in the Supplementary Material.

### Rich-Club Regions

We used the GRETNA toolbox, MATLAB, to analyze and construct the connection matrix for each patient (35). The rich-club coefficient was computed as the average connection weight and represented the density of connections. Then, a rich-club analysis was conducted to identify hubs. Hubs were defined as a degree (k) at least one standard deviation above the network mean (36, 37). Among all nodes in the brain, the top 13 nodes with the highest degree (averaged across all groups) were selected as the rich-club regions (21, 24, 38, 39). The edges between these rich-club regions were defined as rich-club connections (27). We also identified abnormal connections between rich-club nodes. Among rich-club connections, we identified nodes with a higher number of abnormal connections (range = 11–27). BrainNet Viewer toolbox was used to visualize the results (40).

### Relationships Between Network Parameters and Clinical Variables

Pearson's correlations were performed after controlling for age and sex to assess the relationship of structural network properties with cognitive performance and clinical variables. Statistical significance was set at *P* < 0.05.

### Statistical Analysis

SPSS software version 26 (IBM Corp., Armonk, NY, USA) was used for all statistical analyses. The Shapiro-Wilk test was used for testing the data normality (P_ANI_ = 0.268, P_non−HAND_ = 0.152, P_HIV_ = 0.168, and P_HC_ = 0.143). Continuous variables with normal distribution were presented as mean ± standard deviation (SD), and non-normal variables were reported as median (interquartile range). The independent sample *t*-test (for age), the Mann–Whitney *U*-Test (for education), and Fisher's exact test (for sex) were conducted to examine the differences in age, sex, and education between HIV+ patients and healthy controls (HC). Statistical significance was defined at *P* < 0.05.

The differences in topology metrics (at the global and regional levels) of the structural network between groups were discovered using two-sample *t*-tests (HIV+ patients vs. HCs, and ANI group vs. non-HAND group) after correcting for age and sex (*P* < 0.05; when *T*-test was significant), with *post-hoc* tests and Bonferroni corrections for multiple comparisons at *P* < 0.05. Two-sample *t*-tests were used to detect the between-group differences in rich-club connections. The topology metric analysis was performed in the statistical module of GRETNA software. Group differences were also calculated using GRETNA.

## Results

### Demographic, Clinical Data

Table 1 lists the demographic and clinical features of all study participants. There were no significant differences in the clinical data between the groups (*P* > 0.05). The NP test scores for the six cognitive domains in the ANI group tended to be lower than those in the non-HAND group, though this difference did not reach statistical significance (Table 1).

**Table 1:** Demographic and clinical characteristics of the participants.

**Parameters**	**HIV+ (*n* = 48)**	**HC (*n* = 48)**	***P*-Value**	**ANI**	**non-HAND**	***P*-Value**
Sex (men, women)	46/2	44/4	0.677^b^	23/1	23/1	0.100^b^
Age (years; Mean ± SD)	32.25 ± 5.67	34.52 ± 6.52	0.399^a^	31.67 ± 5.48	32.83 ± 5.92	0.418^a^
Education (year; Median ± IQR)	16,16	16,16	0.953^c^	16,16	16,16	0.973^c^
Disease course (months; Mean ± SD)	40.98 ± 31.08			40.54 ± 36.08	41.42 ± 26.71	0.538^a^
Plasma VL (HIV RNA)	TND			TND	TND	
CD4^+^ (Mean ± SD)	586.48 ± 173.13			581.97 ± 169.93	590.99 ± 179.82	0.820^a^
CD4^+^/CD8^+^ratio (Mean ± SD)	0.805 ± 0.50			0.79 ± 0.61	0.82 ± 0.37	0.145^a^
**NP score (Mean** **±SD)**
Verbal fluency	45 ± 8.26			41.25 ± 8.67	48.75 ± 5.91	0.247^a^
Attention/working memory	41.24 ± 7.01			38.08 ± 5.82	44.39 ± 6.76	0.837^a^
Executive function	54.72 ± 11.04			49.64 ± 10.75	59.79 ± 8.93	0.275^a^
Learning/delayed recall	43.11 ± 7.71			39.10 ± 6.83	47.13 ± 6.43	0.657^a^
Speed of information processing	44.09 ± 9.19			40.35 ± 8.25	47.83 ± 8.68	0.805^a^
Fine motor skills	43.65 ± 10.23			40.40 ± 11.60	46.90 ± 7.58	0.082^a^

*The comparison of age and neuropsychological scores between groups were done using an independent t-test. Gender differences were conducted by Fisher‘s exact test. Education differences were conducted by the Mann–Whitney U-Test. Significance was set as P < 0.05. NP, neuropsychological performance; VL, viral load; HIV+, human immunodeficiency virus positive; HC, healthy control; ANI, asymptomatic neurocognitive impairment; non-HAND, non-HIV-associated neurocognitive disorders. ^a^t-test; ^b^Fisher‘s exact test; ^c^Mann–Whitney U-Test; SD-standard deviation*.

### Global Network Properties

The global properties of HIV+ patients were weaker than those of HC. Although significant alterations were expected in the global features of those with HIV, we found no significant changes between HIV+ patients and HCs and between non-HAND and ANI groups. Furthermore, HIV+ patients showed no small-worldness characteristic.

### Nodal Network Properties

**Figure 1:**
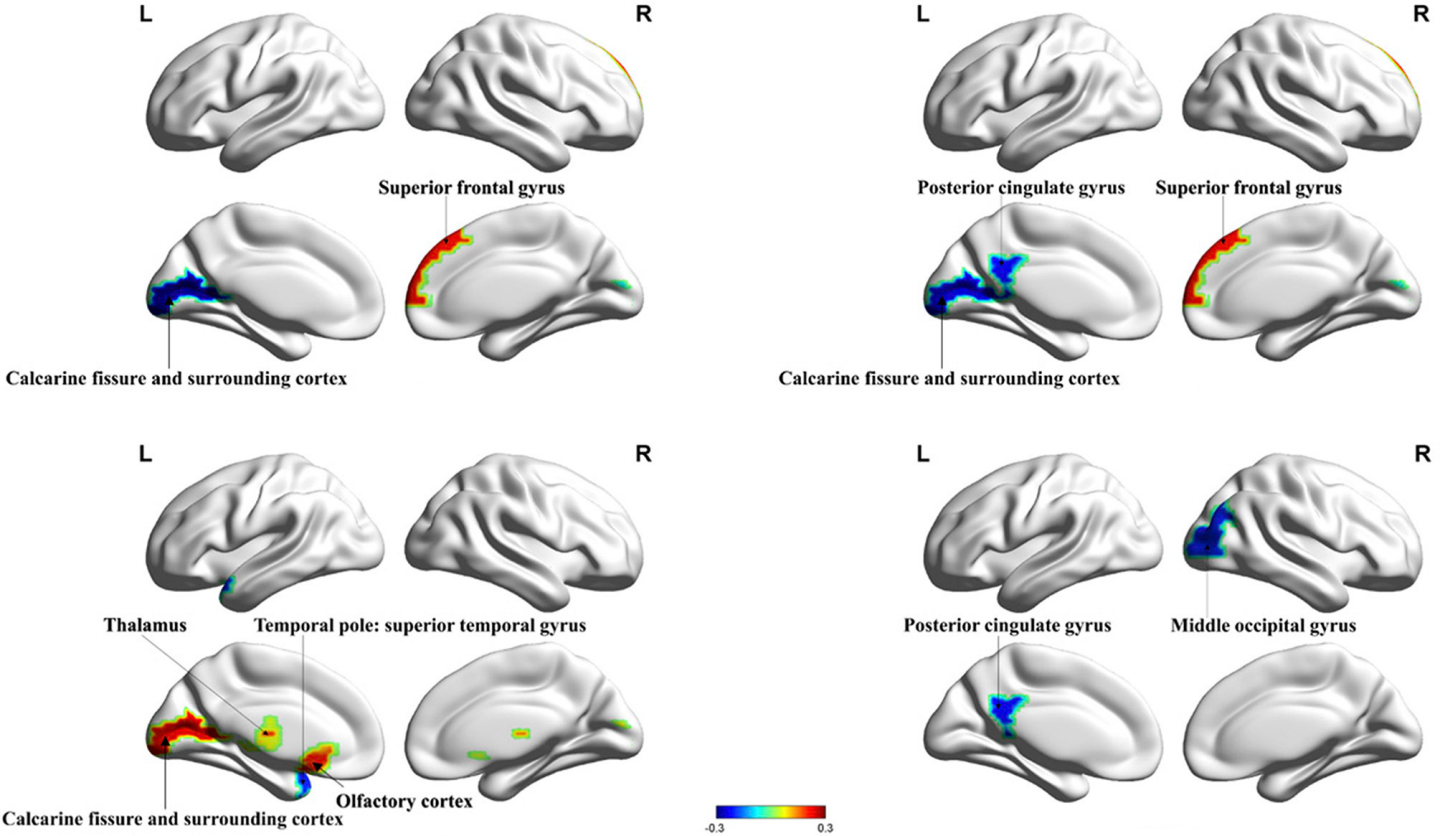
Significant alterations in the nodal properties (*P* < 0.05) within whole brain structural network analysis between HIV+ patients and healthy controls are projected onto both hemispheres of the brain surface and over a range of thresholds, as shown. Hot colors represent brain regions with increased nodal properties, whereas cool colors represent decreased nodal properties. Betweenness centrality was significantly decreased in the calcarine fissure and surrounding cortex and significantly increased in the superior frontal gyrus. Degree centrality was significantly decreased in the posterior cingulate gyrus, the calcarine fissure, and the surrounding cortex and increased in the superior frontal gyrus. The nodal cluster coefficient in the temporal pole was significantly decreased in the superior temporal gyrus and increased in the calcarine fissure and surrounding cortex, thalamus, and olfactory cortex. Nodal efficiency in the posterior cingulate gyrus and middle occipital gyrus were significantly decreased. BC, betweenness centrality; DC, degree centrality; NCE, nodal cluster coefficient; NE, nodal efficiency.

**Figure 2:**
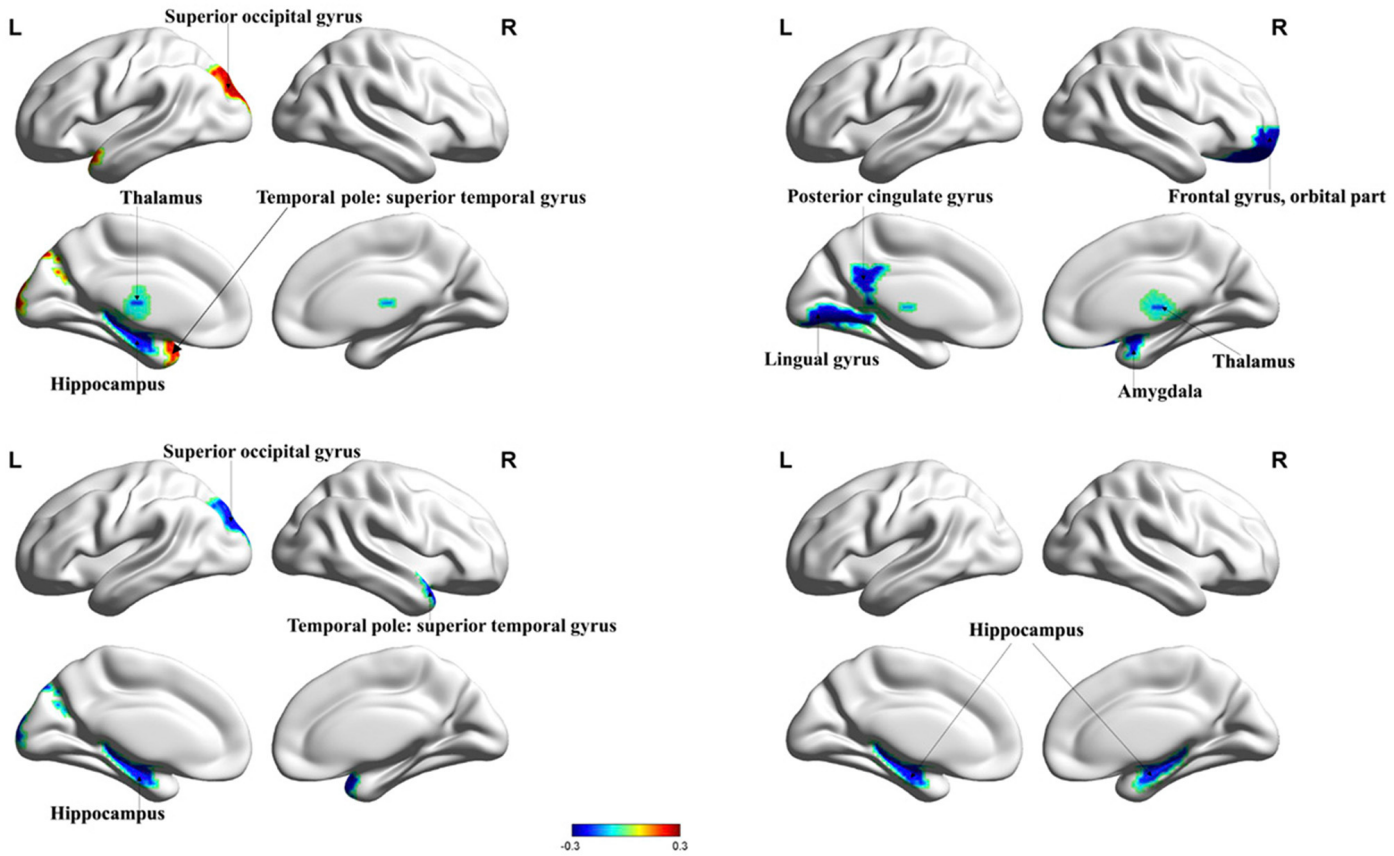
Significant changes in the nodal properties (*P* < 0.05) within different brain regions between the ANI and non-HAND groups are projected onto both hemispheres of the brain and over a range of thresholds, as shown. Hot colors represent brain regions with increased nodal properties, whereas cool colors represent decreased nodal properties. The betweenness centrality was significantly decreased in the hippocampus and thalamus and significantly increased in superior occipital gyrus and temporal pole: superior temporal gyrus. The degree of centrality was significantly decreased in the lingual gyrus, posterior cingulate gyrus, frontal gyrus, orbital part, amygdala, and thalamus. The nodal cluster coefficient was significantly decreased in the superior occipital gyrus, temporal pole: superior temporal gyrus, and hippocampus. Nodal efficiency in the hippocampus was significantly decreased. BC, betweenness centrality; DC, degree centrality; NCE, nodal cluster coefficient; NE, nodal efficiency.

HIV+ patients demonstrated decreased degree centrality, betweenness centrality, and nodal efficiency at the occipital lobe and the limbic cortex. We also found increased BC and nodal cluster coefficient in the occipital lobe, the frontal lobe, the insula, and the thalamus. The ANI group demonstrated a significant reduction in degree centrality, nodal clustering coefficient, and nodal efficiency in widespread brain regions encompassing the occipital lobe, the frontal lobe, the temporal pole, the limbic system, the putamen, the thalamus, and the hippocampus. Increased BC was also seen in the superior occipital gyrus and the temporal pole (Figures 1, 2 and Table 2).

**Table 2:** Comparison of structural network nodal properties in all nodes among HIV+ patients and HC groups and ANI and non-HAND groups.

**Nodal properties**	**HIV and HC**			**ANI and non-HAND**		
	**Brain regions**	***t* value**	***P*-Value**	**Brain regions**	***t* value**	***P*-Value**
BC	SFGmed.R	2.1727	0.0324	SOG.L	2.5943	0.0129
	CAL.L	−2.9246	0.0044	TPOsup.L	2.0366	0.0479
	PUT.L	2.2571	0.0264	HIP.L	−2.1556	0.0368
				THA.L	−2.1406	0.0380
DC	SFGmed.R	2.1975	0.0305	ORBsup.R	−2.4388	0.0189
	PCG.L	−2.2146	0.0293	ORBmid.R	−2.6177	0.0122
	CAL.L	−2.5370	0.0129	PCG.L	−2.4469	0.0186
				AMYG.R	−2.2814	0.0275
				LING.L	−2.3648	0.0226
				THA.R	−2.0456	0.0469
NCE	OLF.L	2.1089	0.0377	HIP.L	−2.1086	0.0408
	CAL.L	2.4438	0.0165	SOG.L	−2.1246	0.0394
	THA.L	2.1540	0.0339	PUT.L	−2.1444	0.0377
	TPOsup.L	−2.0127	0.0471	TPOsup.R	−2.2234	0.0315
NE	PCG.L	−2.2005	0.0303	HIP.L	−2.1505	0.0372
	MOG.R	−2.0231	0.0460	HIP.R	−2.0183	0.0498

*A positive t-value represents increased nodal properties, whereas a negative t-value represents decreased nodal properties. BC, betweenness centrality; DC, degree centrality; NCE, nodal cluster coefficient; NE, nodal efficiency. The significance threshold was set at P < 0.05. The abbreviations of brain regions are given in online Supplementary Materials*.

### Rich-Club and Abnormal Connections

**Figure 3:**
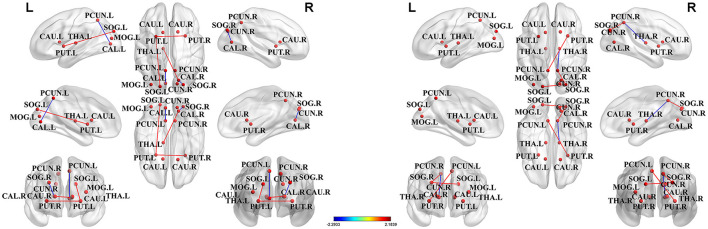
The hub nodes of structural network across the group. Red dots represented the brain hub nodes of the brain, lines between nodes represented the abnormal connections between hub nodes, red lines indicated the enhancement connection, and blue lines indicated the reduced connection. PCUN, precuneus; SOG, superior occipital gyrus; MOG, middle occipital gyrus; CAL, calcarine fissure and surrounding cortex; PUT, putamen; CAU, caudate nucleus; THA, thalamus; CUN, cuneus.

Among all nodes across the groups, we identified 13 nodes with the highest degree (based on the averaged nodal degree) that were a part of the hubs. Among HIV+ patients and the HC group, the rich-club regions were (as sorted by degree) as follows: the putamen (right and left), the precuneus (right and left), the calcarine fissure and the surrounding cortex (right), the caudate nucleus (right), the thalamus (left), the superior occipital gyrus (right), the calcarine fissure and the surrounding cortex (left), the superior occipital gyrus (left), the caudate nucleus (left), the middle occipital gyrus (left), and the cuneus (right). Among ANI and non-HAND groups, the rich-club regions were (sorted by degree) as follows: the putamen (right and left), the precuneus (right and left), the calcarine fissure and the surrounding cortex (right), the thalamus (left), the caudate nucleus (right), the superior occipital gyrus (left and right), the middle occipital gyrus (left), the caudate nucleus (left), the cuneus (right), and the thalamus (right). Furthermore, these hub nodes were located in the resting state network. The cuneus, the calcarine fissure and the surrounding cortex, the superior occipital gyrus, and the middle occipital gyrus were located in the visual network. The putamen and the caudate nucleus were located in the salience network (Table 3 and Figure 3).

**Table 3:** Hub regions among pairs of comparing groups (sorted by degree).

**Number**	**HIV+ patients and HC**	**ANI and Non-HAND**
1	PUT.R	PUT.R
2	PUT.L	PUT.L
3	PCUN.R	PCUN.R
4	PCUN.L	PCUN.L
5	CAL.R	CAL.R
6	CAU.R	THA.L
7	THA.L	CAU.R
8	SOG.R	SOG.L
9	CAL.L	SOG.R
10	SOG.L	MOG.L
11	CAU.L	CAU.L
12	MOG.L	CUN.R
13	CUN.R	THA.R

Hub nodes were sorted by degree as follows, node number 1 represents the node with the highest degree, whereas node 13 represents the node with the least degree among hub nodes. Abbreviations for brain regions are listed in (Supplementary Table 1).

**Figure 4:**
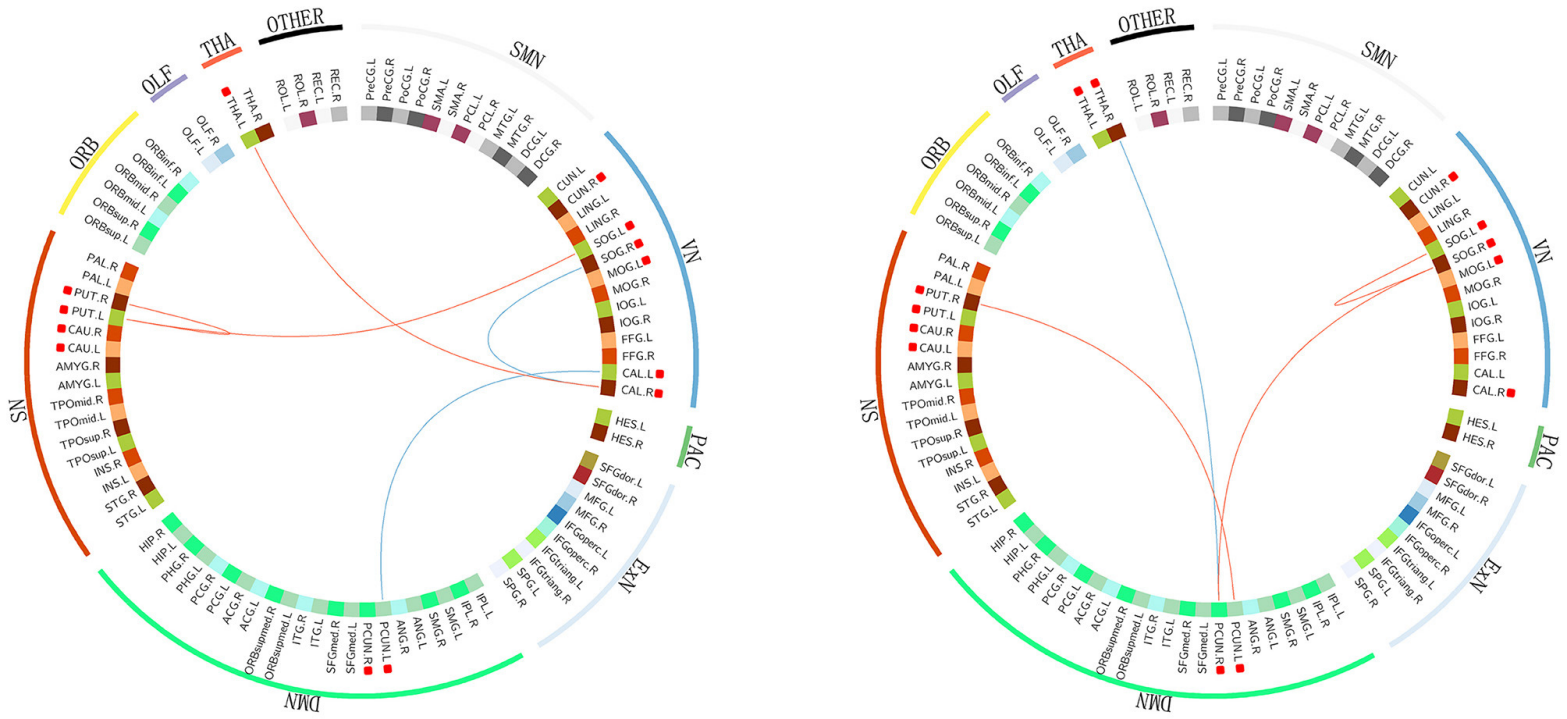
The abnormal rich-club connections are shown in functional networks: red edges denote a positive T score, representing the enhancement of the connection. Blue edges denote a negative T score, representing a decrease in the connection. Hub nodes are highlighted with red square boxes in front. On comparing the HIV+ group with HC, increased connections were seen between the left thalamus and the visual network, between the salience and visual networks, and in intra-connection within the salience network. Decreased connections were seen between the visual and default mode networks and in intra-connection within the visual network. On comparing the ANI group with the non-HAND group, increased connections were seen between the visual network and the default mode network, between the salience and default mode networks, and in intra-connection within the visual network. Decreased connections were seen between the right thalamus and the default mode network. The structures located in the visual network included the cuneus, the calcarine fissure and the surrounding cortex, the superior occipital gyrus, and the middle occipital gyrus. Those in the salience network included the putamen and caudate nucleus. DMN, default mode network; SN, salience network; VN, visual network; SMN, sensory motor network; PAC, primary auditory cortex; ExN, executive network; ORB, orbital frontal cortex; OLF, olfactory cortex; THA, thalamus. PCUN, precuneus; SOG, superior occipital gyrus; MOG, middle occipital gyrus; CAL, calcarine fissure and surrounding cortex; PUT, putamen; CAU, caudate nucleus; THA, thalamus; CUN, cuneus.

The abnormal rich-club connections were identified and compared in both groups. On comparing the HIV+ group with HCs, connections between the left putamen and the superior occipital gyrus, the left putamen and the right putamen, and the left thalamus and the right calcarine fissure were all enhanced. In contrast, connections between the left calcarine fissure and the posterior cingulate gyrus and between the right calcarine fissure and the superior occipital gyrus were decreased. On comparing the ANI group with the non-HAND group, connections between the right putamen and the left posterior cingulate gyrus, between the right posterior cingulate gyrus and the right superior occipital gyrus, and between the left and right superior occipital gyrus were enhanced. In contrast, connections between the right thalamus and the right posterior cingulate gyrus were decreased. We located these abnormal rich-club connections in the resting state network. Furthermore, intra-connections (within the network) and inter-connections (between other networks) were also discovered. On comparing the HIV+ group with HCs, increased connections were seen between the left thalamus and visual network, between the salience network and the visual network, and in intra-connections within the salience network. Decreased connections were seen between the visual network and the default mode network and in intra-connections within the visual network. On comparing the ANI group with the non-HAND group, increased connections were seen between the visual network and the default mode network, between the salience network and the default mode network, and in intra-connections within the visual network. Decreased connections were also seen between the right thalamus and the default mode network (Figure 4).

### Correlation Between Connectivity Metrics and Cognitive Performance, Clinical Variables

We found that the disease course was negatively correlated with nodal efficiency in the middle occipital gyrus (*r* = −0.29; *P* = 0.04). Degree centrality in the superior frontal gyrus was negatively correlated with CD4 counts (*r* = −0.35; *P* = 0.01), and the structural connection between the left putamen and the right putamen was negatively correlated with CD4/CD8 ratio (*r* = −0.36; *P* = 0.01). Further, BC in the calcarine fissure was positively correlated with complex motor skills (*r* = 0.32; *P* = 0.03).

## Discussion

In this study, HIV+ patients with successful plasma viral suppression were found to have structural connectivity alterations at the regional level, mainly in the occipital lobe and the subcortical regions, along with cognitive impairment. We also found that nodal connectivity metrics in ANI patients were consistently decreased. These results did not survive the Bonferroni correction. We identified 13 brain regions as hub nodes, mainly located in the occipital lobe and the subcortical regions, and they remained stable in both groups. The abnormal connections were mainly located in the occipital lobe in the HIV+ group and in the parietal lobe in the ANI group. After we sorted the brain regions into RSN, the abnormal rich-club connections in HIV+ patients were mainly found in the visual network and in ANI patients in the default mode network. The BC in the calcarine fissure was positively correlated with complex motor skills, and the disease course was negatively correlated with nodal efficiency in the middle occipital gyrus. Overall, these findings suggest that the occipital lobe and the subcortical regions may play important role in the structural connectivity in HIV+ patients with fully suppressed plasma viral loads.

A major discovery of our study was that structural network alterations in the occipital lobe appeared in several properties. The centrality and nodal efficiency of the occipital lobe were both decreased. The reduction of centrality indicated that the number of shortest path lengths through the occipital lobe was decreased, meaning that its role in transmitting information was diminished. Nodal efficiency reduction indicates the impairment of associated brain regions, and the reduction of these connectivity properties in our study indicated that white matter in the occipital lobe was damaged, and its significance in the structural network was compromised. This is in line with previous studies where patients with HIV have shown a significant neuronal loss of up to 30% in the calcarine cortex (41). The occipital lobe may be more vulnerable to neurotoxicity (42), and cerebral blood flow in the visual cortex may be reduced in those with chronic HIV infection, leading to atrophy of the occipital lobe (43). The white matter in the parieto-occipital region has been shown to be particularly vulnerable to HIV infection (44). Our previous research showed lower spontaneous brain activity and volume reduction in the occipital lobe (45, 46). We assume that, once HIV penetrates into the CNS, the neurons and white matter fibers in the occipital lobe may be damaged before those in other brain regions. The redistribution of structural networks occurs in order to preserve normal cognition, mediated by viral reservoirs suppressing immune response in the occipital lobe (47). In HIV+ patients, the occipital lobe is associated with visual attention, visual working memory, and episodic memory (48). In this study, HIV+ patients had lower scores on the visuospatial memory test, the Wisconsin card sorting test, and the grooved pegboard test, and ANI patients scored lower than non-HAND patients. In the correlation analysis, we found that BC in the calcarine fissure positively correlated with complex motor skills. On the same note, previous research reported that occipital volume reduction was correlated with complex motor skills (49). Meanwhile, the disease course was negatively correlated with nodal efficiency in the middle occipital gyrus, which suggests a compromise of the middle occipital gyrus with prolongation of the disease course.

Another important finding of this study was that of significant structural network alterations in the subcortical regions. Subcortical structures are a group of brain regions located deep in the brain; they are not only involved in complex cognitive activities but also act as information hubs for the relay and regulation of information through different areas of the brain. In the pre-cART era, due to the appearance of motor, attention, and memory dysfunction (50–52), HAND was considered subcortical dementia (53). In advanced stages of HAND, basal ganglia may have higher viral loads and volume reduction (54–56). In our study, nearly all structural features in the subcortical area were decreased, which indicates a possible compromise in their ability to transmit information. Prior research has shown a similar finding of subcortical damage in virologically-suppressed cohorts (57). We detected alterations in the early stages, which may suggest that the subcortical regions remain vulnerable despite successful cART. Another structural connectivity study on pediatric HIV+ patients similarly showed subcortical volume reduction (26). The subcortical regions are considered to be related to cognitive functions such as memory and attention. In this study, HIV+ patients had poorer NP test scores in attention/working memory, learning, and delayed recall. Compared with non-HAND patients, ANI patients performed poorer in these aspects. A previous study showed that, in the post-cART era, HAND remains subcortical (58). Therefore, we believe that subcortical regions may play an important role in the early diagnosis of HAND. The structural connectivity analysis may be a sensitive tool for detecting HAND. Patients in this study have several characteristics, all patients were under successful cART, and viral load in plasma was fully suppressed. We believe that early and effective treatment may have significant benefits on cognitive impairment. Since the average age of patients was relatively young and patients may not have reached the age that could trigger the significant alteration, we believe that a longitudinal study will give us more information. Finally, patients in this study were either in normal cognition or in the early stages of HAND. We believe that these could be reasons why the connectivity metrics in this study did not survive the Bonferroni correction. However, we still believe that the results are valuable for HAND research. Our results showed that, even with successful treatment, patients were still having subtle changes in the brain. With the growing population of HIV+ patients with suppressed plasma viral load, understanding the topological alterations in these patients could contribute to revealing the neuropathology of HAND, although the alterations may not be significant. Furthermore, based on our results, there should be more research on finding sensitive techniques to reveal the structural changes. We assume that there will be more significant results from longitudinal research with a larger cohort.

Brain networks provide us with a new perspective to understand the brain as a whole, instead of studying it as a group of isolated brain regions. This study throws light on connections between brain regions that are structurally distant. Rich-club is considered the fundamental property that ensures efficient transfer of information and is the basis of complex neurological functions (24). In this study, rich-club regions were identified across groups (27, 39), and we identified 13 hub nodes, mostly located in the subcortical regions and the occipital lobe, particularly in the thalamus and the basal ganglia. Hub distribution was similar among the HIV+ and ANI groups, indicating that hub nodes are relatively stable in HIV+ patients, even those with pre-clinically cognitive impairment. Our results were in line with previous research on functional connectivity (59, 60), showing that the hub regions overlapped between groups and were re-distributed on the deterioration of cognition. We found no hub nodes in the frontal and temporal lobes in this study, and we are unable to explain this phenomenon at this time; therefore, a rich-club analysis should be conducted in a larger cohort of patients, and a longitudinal study would be ideal to monitor changes in hub nodes. Nevertheless, by understanding rich-club, we could reveal how brain damage leads to cognitive disorders and were able to assess cognitive impairment using a more efficient method. Potentially, this may contribute to new treatments that can target certain brain regions and prevent further cognitive impairment.

We also investigated abnormal rich-club connections in this study. We observed that these abnormal connections were not simply decreased, as several connections were also increased, suggesting that the brain could regulate connections among brain regions to optimize cognitive performance. Studies have proposed the “hub overload and failure” theory (61, 62), which states that redistribution of the network passage in damaged regions into other passages of higher connection eventually leads to the failure of hub regions. The enhancement of connections in this study may suggest that the overload starts at the rich-club nodes, but further longitudinal research is needed to validate this. We divided the brain regions with RSN parcellation and aimed to identify connections between functional and structural connectivity. We found that most abnormal connections in HIV+ patients were located in the VN, and the ANI group showed the most abnormal connections in the DMN. These results are consistent with the finding of our previous study (46). Wang et al. (63) also reported prominent changes in visual networks in the early stages of HIV infection. Other studies on HAND have also reported DMN alterations (64–66). As far as we know, this is the first study to focus on abnormal connections in the structural network in an HIV+ cohort. Further research into abnormal connections in HIV+ patients can reveal details on structural alterations and combining the functional and structural analyses in these patients can help us understand the neuropathology of HAND comprehensively.

We found that not all structural connectivity alterations were decreased; on comparing HIV+ patients with HCs, the centrality and nodal cluster coefficient were increased in the occipital lobe, the frontal lobe, the insula, and the thalamus. Furthermore, no significant alterations were discovered at the global level. Previous studies investigated patients with no prior treatment history or those who were mostly treated, and their results were mainly reflected at the global level (19, 20). The patients in this study were all on successful cART and their plasma viral load was undetectable. We believe that early cART treatment is beneficial in protecting against cognitive impairment. Although the legacy effect (67), drug toxicity (68), and regional inflammation were all reported as possible contributors to persistent neuronal damage in HIV+ patients, Boban et al. (69) also stated that cART may be inadequate in preventing the neurodegenerative process. Nevertheless, currently, cART is still the best treatment available. A previous study concluded that those with complete viral suppression in the plasma had better neurocognition than those with detectable viral loads (70). Studies have also suggested that initiation of cART in the early stage may contribute to long-term brain health (28, 71). The brain most likely reorganizes connectivity distributions to ensure stable cognition. Thus, increased connectivity metrics may be caused by redistribution or a compensatory effect. Future longitudinal studies are warranted to explore this further.

This study is not without limitations. First, the sample size was relatively small. Although there was a sufficient number of patients compared with previous studies, a larger sample size might avoid bias due to individual differences. Second, most of our participants were men; a similarly skewed sex ratio has been reported in several studies, and there should be greater female involvement in future studies. Third, this was a cross-sectional study; longitudinal analysis might reveal alterations more objectively. Fourth, the structural connectivity results did not survive the Bonferroni corrections; a larger cohort including patients with different cognitive statuses should be studied in the future. Finally, NP tests were not employed on HCs; future studies should conduct prior NP tests on HCs for reliable comparisons.

## Conclusion

We demonstrated the structural connectivity alterations, hub regions, and abnormal rich-club connections in HIV+ patients with fully suppressed viral loads. The findings of this study suggested that the occipital lobe and the subcortical regions play an important role in structural changes and cognitive impairment. Hubs and abnormal rich-club connections may contribute to our understanding of the neuropathology of HAND. However, rich-club studies in HIV+ patients are not sufficient; more research should be conducted in the future. Our study demonstrated new results for HIV+ patients, and these results may contribute to finding new neuroimaging biomarkers and understanding the pathogenesis of HAND.

## Data Availability Statement

The raw data supporting the conclusions of this article will be made available by the authors, without undue reservation.

## Ethics Statement

The studies involving human participants were reviewed and approved by Beijing Youan Hospital Ethics Committee. The patients/participants provided their written informed consent to participate in this study.

## Author Contributions

XA had full access to all the data in the study and took responsibility for the integrity of the data, the accuracy of the data analysis, and draft of the manuscript. RL and HL study concept and design. XA, WW, AZ, ZJ, and XL acquisition of data. BR and XA analysis and interpretation of data. RL, BR, and HL critical revision of the manuscript for important intellectual content and study supervision. WW, AZ, and ZJ statistical analysis. All authors read and approved the final manuscript.

## Funding

This work was supported by the National Natural Science Foundation of China (grant nos. 81771806 and 61936013), Beijing Natural Science Foundation (7212051), Capital Medical University research and incubation funding (grant no. PYZ19162), Beijing Excellent Talent Plan (grant no. 2018000021469G290), Beijing Municipal Health Commission, Technology Achievements and Appropriate Technology Promotion Project (grant no. 2020-TG-002), and You'an Medical Development Project of COVID-19 Emergency Prevention and Control Public (grant no. BJYAYY-2020YC-03).

## Conflict of Interest

The authors declare that the research was conducted in the absence of any commercial or financial relationships that could be construed as a potential conflict of interest. The reviewer LL declared a shared affiliation with the Author BR to the handling editor at the time of review.

## Publisher's Note

All claims expressed in this article are solely those of the authors and do not necessarily represent those of their affiliated organizations, or those of the publisher, the editors and the reviewers. Any product that may be evaluated in this article, or claim that may be made by its manufacturer, is not guaranteed or endorsed by the publisher.
