# Supplementary material for: Rich-Club Analysis of Structural Brain Network Alterations in HIV Positive Patients With Fully Suppressed Plasma Viral Loads
Source: Front Neurol. 2022 Jun 24;13:825177. doi: 10.3389/fneur.2022.825177 (PMC9263507; doi:10.3389/fneur.2022.825177)
Supplement: Supplementary file 1 [file Data_Sheet_1.PDF]

Supplementary Material

2.5. Graph theory

2.5.1 Global properties of the network

On the global level, global clustering coefficient: symbols of interconnectivity level in the network, manifest as average clustering coefficients of all nodes, and it is the fractional value of dividing the actual number of edges by the maximum number of possible edges. Characteristic path length represents the network information transmission, lower this indicator it is, higher the transmission efficiency, the least edges connecting two nodes is defined as the path length of two nodes, and the mean value of the path length of all node pairs in the network is defined as the characteristic path length. Global efficiency is the parameters that could indicate global transmission efficiency of the network. The global efficiency is defined as the reciprocal of the harmonized average of the shortest path length between all node pairs; connection strength: the sum of all nodal connections; local efficiency: demonstrate the network interconnection, showing as the inverse of shortest path length to measures the efficiency of network transferring information.

Comparing the random networks with real networks, we can analyze the small-worldness. There are three critical characteristics to establish the small-world network, gamma (ratio of the cluster coefficient), lambda (ratio of the shortest path length), and sigma (ratio of gamma and lambda). Noticeably, gamma and lambda are the ratios of real network and random network, and sigma guarantees the transmission efficiency at both global and local levels. It has a high clustering coefficient as well as the shortest path length.

2.5.2 Regional nodal network characteristics

On the regional level, we analyzed nodal degree: influence of certain nodes in the whole network, the sum of edges directly connected to a node is defined as the degree of that node; nodal betweenness centrality: illustrating the importance of certain nodes to network information processing capacity, all the shortest pathways that pass through certain nodes; nodal efficiency: important parameter that could identify brain regions with impaired white matter, A node with high nodal efficiency indicates great interconnectivity with other regions in the network.

Results

3.3. Nodal properties of network

There were significant changes in regional level, between the HIV+ subjects and the HC group, HIV+ subjects had a reduced betweenness centrality in left calcarine fissure and surrounding cortex (t=-2.9246, p=0.0044), whereas increased betweenness centrality in left putamen (t=2.2571, p=0.0246) and right superior frontal gyrus, medial (t=2.1727, p=0.0324). Furthermore, HIV+ subjects had a reduced degree centrality in left posterior cingulate gyrus (t=-2.2146, p=0.0293) and left calcarine fissure and surrounding cortex (t=-2.5370, p=0.0129), whereas an increased in right superior frontal gyrus, medial (t=2.1975, p=0.0305). For nodal clustering coefficient, HIV+ subjects showed decreased in left temporal pole: superior temporal gyrus (t=-2.0127, p=0.0471), increased in left thalamus (t=2.1540, p=0.0338), left calcarine fissure and surrounding cortex (t=2.4439, p=0.0164), olfactory cortex (t=2.1089, p=0.0377). HIV+ subjects showed deceased nodal efficiency in left posterior cingulate gyrus (t=-2.2010, p=0.0303) and middle occipital gyrus (t=-2.0231, p=0.0460).

Between the ANI and Non-HAND group, ANI subjects showed decreased betweenness centrality in left hippocampus (t=-2.1556, p=0.0367) and left thalamus (t=-2.1406, p=0.0380), whereas increased in left superior occipital gyrus (t=2.5944, p=0.0479) and left temporal pole: superior temporal gyrus (t=2.0366, p=0.0479). ANI group showed consistency in degree centrality reduction, mainly in thalamus (t=-2.4388, p=0.0189), lingual gyrus (t=-2.3648, p=0.0226), amygdala (t=-2.2814, p=0.0275), posterior cingulate gyrus (t=-2.4469, p=0.0186), middle frontal gyrus- orbital part (t=-2.6177, p=0.0121), superior frontal gyrus-orbital part (t=-2.4388, p=0.0189). Moreover, ANI group showed reduction in nodal cluster coefficient: left hippocampus (t=-2.1087, p=0.0408), left superior occipital gyrus (t=-2.1246, p=0.0394), left putamen (t=-2.1443, p=0.0377), temporal pole: superior temporal gyrus (t=-2.2234, p=0.0315). ANI group showed reduction in nodal efficiency as well: left hippocampus (t=-2.1505, p=0.0372), right hippocampus (t=-2.0183, p=0.0499). Furthermore, we did not find significant between-group difference in nodal shortest path length. There are no results showed significant difference under the FDR correction.

Supplementary Tables

Supplementary Table 1. The abbreviations of the 90 brain regions in AAL-90 atlas

Number	Brain region	abbreviations
1,2	Precentral gyrus	PreCG
3,4	Superior frontal gyrus, dorsolateral	SFGdor
5,6	Superior frontal gyrus, orbital part	ORBsup
7,8	Middle frontal gyrus	MFG
9,10	Middle frontal gyrus, orbital part	ORBmid
11,12	Inferior frontal gyrus, opercular part	IFGoperc
13,14	Inferior frontal gyrus, triangular part	IFGtriang
15,16	Inferior frontal gyrus, orbital part	ORBinf
17,18	Rolandic operculum	ROL
19,20	Supplementary motor area	SMA
21,22	Olfactory cortex	OLF
23,24	Superior frontal gyrus, medial	SFGmed
25,26	Superior frontal gyrus, medial orbital	ORBMed
27,28	Gyrus rectus	REC
29,30	Insula	INS
31,32	Anterior cingulate and paracingulate gyri	ACG

33,34	Median cingulate and paracingulate gyri	MCG
35,36	Posterior cingulate gyrus	PCG
37,38	Hippocampus	HIP
39,40	Para hippocampal gyrus	PHG
41,42	Amygdala	AMYG
43,44	Calcarine fissure and surrounding cortex	CAL
45,46	Cuneus	CUN
47,48	Lingual gyrus	LING
49,50	Superior occipital gyrus	SOG
51,52	Middle occipital gyrus	MOG
53,54	Inferior occipital gyrus	IOG
55,56	Fusiform gyrus	FFG
57,58	Postcentral gyrus	PoCG
59,60	Superior parietal gyrus	SPG
61,62	Inferior parietal lobule	IPL
63,64	Supramarginal gyrus	SMG
65,66	Angular gyrus	ANG
67,68	Precuneus	PCUN
69,70	Paracentral lobule	PCL
71,72	Caudate	CAU

73,74	Putamen	PUT
75,76	Pallidum	PAL
77,78	Thalamus	THA
79,80	Heschl gyrus	HES
81,82	Superior temporal gyrus	STG
83,84	Temporal pole (Superior)	TPOsup
85,86	Middle temporal gyrus	MTG
87,88	Temporal pole (Middle)	TPOmid
89,90	Inferior temporal	ITG

Odd numbers represent brain regions on the left hemisphere, even numbers represent brain regions on the right hemisphere.
